# Community-Based Control of a Neglected Tropical Disease: The Mossy Foot Treatment and Prevention Association

**DOI:** 10.1371/journal.pntd.0000424

**Published:** 2009-05-26

**Authors:** Gail Davey, Emily Burridge

**Affiliations:** 1 School of Public Health, Addis Ababa University, Addis Ababa, Ethiopia; 2 St George's Hospital Medical School, London, United Kingdom; University of Buea, Cameroon

## Introduction

Podoconiosis (endemic non-filarial elephantiasis, also known as mossy foot) is a non-communicable disease now found exclusively in the tropics, caused by the conjunction of environmental, genetic, and economic factors. Silicate particles formed by the disintegration of lava in areas of high altitude (over 1,000 m) and seasonal rainfall (over 1,000 mm per annum) penetrate the skin of barefoot subsistence farmers, and in susceptible individuals cause lymphatic blockage and subsequent elephantiasis [1]. Although an estimated one million Ethiopians (of a total population of 77 million) are afflicted with podoconiosis [2], which creates a huge economic burden in endemic areas [3], no national policy has yet been developed to control or prevent the condition, and most affected communities remain unaware of treatment options.

## The Mossy Foot Treatment and Prevention Association

The Mossy Foot Treatment and Prevention Association (MFTPA) is an Ethiopian non-governmental organization founded in 1998 and registered with the Ethiopian Ministry of Justice in 2000. From inception, its mission has been the treatment and prevention of podoconiosis. The MFTPA is based in Wolaita zone, southern Ethiopia, an area of 1.7 million inhabitants known to bear a heavy podoconiosis burden [2], and was founded by health professionals who were concerned by the lack of treatment available for patients, and the reaction patients met when trying to access government services in this highly endemic area. From a modest beginning treating 1,000 patients per year at one central site, the organization has developed so that 35,000 patients are now treated annually at 15 outreach sites.

The MFTPA is organized into clinical, social work, and administration sections. At present, clinics offering health education and treatment are held weekly at 15 sites located between 15 and 65 km of the headquarters in Sodo town, southern Ethiopia. Clinics are run by community podoconiosis agents (CPAs) who are themselves patients, and social workers who are recruited from the local community. Both these groups of workers are paid a monthly salary by the MFTPA. CPAs are managed by the project director and social workers by the head of social work, both of whom visit at least each fortnight to supervise and replenish supplies of treatment consumables.

The project director is also responsible for initiating and monitoring network groups. These groups are made up of local leaders from government offices, schools, churches, and women's groups. Network group participants are unpaid, and take on the role of promoting “community conversations” after receiving training about the disease and its control. Quarterly meetings of CPAs, social workers, and network group representatives are held in Sodo town, and include in-service training, feedback of performance evaluation, and joint strategy planning.

Most funding for the MFTPA comesfrom the Mossy Foot Project, a United States–registered fund-raising organization. A relatively small proportion comes from within Ethiopia, through project grants and donations. The Ethiopian Federal Ministry of Health contributes “in kind” through use of the grounds and storage areas of government health clinics for MFTPA clinic sites.

The Ministry of Health has encouraged the MFTPA to take a national role in prevention and control of podoconiosis by expanding to podoconiosis-endemic areas in other parts of Ethiopia. Before expansion, evaluation of the organizational structure of the program was recommended. This article documents qualitative review of the MFTPA's delivery of podoconiosis care against a model framework. This is therefore not an evaluation of efficacy or effectiveness of a specific intervention (unlike those performed in relation to filarial disease [4],[5]), but a program evaluation intended to identify weak program areas.

## Development of the Innovative Care for Chronic Conditions Framework

**Figure 1:**
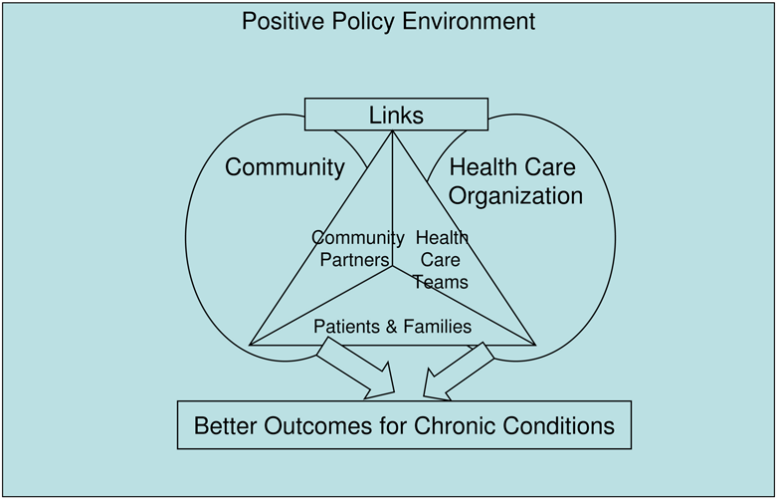
The ICCC framework. Adapted from [6].

The Innovative Care for Chronic Conditions (ICCC) framework [6] was developed by a working group of the World Health Organization in response to the burden placed on developing countries' health care systems by non-communicable diseases. The ICCC framework evolved out of the Chronic Care Model, which was developed to enable primary care practices in developed country settings to improve care for patients with chronic disease [7]. The authors describe the ICCC framework as “comprised of fundamental components within the patient (micro-), organization/community (meso-), and policy (macro-) levels. These components are described as ‘building blocks’ that can be used to create or re-design a health care system that can more effectively manage long-term health problems” [8]. The framework is represented figuratively in both articles describing its development and use (Figure 1). Compared to the Chronic Care Model, the ICCC framework emphasizes the importance of the community and community partners, and the necessity of a positive policy environment [8].

## Selection of the ICCC Framework for Evaluation of the MFTPA

Epping-Jordan and colleagues cite examples of use of the ICCC framework as a conceptual basis for several chronic disease programs in Morocco, the Russian Federation, and Rwanda [8]. Several subsequent articles have contextualized the ICCC framework among other chronic disease care systems, and a few in low-income settings have focused on evaluating single components rather than the whole system (including, for example, self-care in leprosy patients [9]). We selected the ICCC framework because of its “building blocks” approach, reasoning that it would enable us to qualitatively identify areas of the program that were deficient and required strengthening. We were unable to identify another framework so well suited to evaluation of a chronic disease program in a resource-poor setting.

## Methods

We broke down the ICCC framework by level and then by component (Table 1, first three columns). We next identified the individuals or groups likely to act as sources of information relating to each component (Table 1, fourth column). The ICCC framework does not include quantitative elements by which to evaluate each component, so we decided simply to collect narrative information around each component.

**Table 1:** ICCC Framework by Level and Component.

Level	Component	Characteristic or Function	Information Source
**Micro**	Patients and families	Informed	Patients and families
		Prepared	
		Motivated	
	Community partners	Informed	Network groups
		Prepared	
		Motivated	
	Health care team	Informed	CPAs
		Prepared	
		Motivated	
**Meso**	The community	Raise awareness and reduce stigma	Network groups
		Encourage better outcomes through leadership and support	
		Mobilize and coordinate resources	
		Provide complementary services	
	The health care organization	Promote continuity and coordination	Project director; social work director; administrative staff; record review
		Encourage quality through leadership and incentives	
		Organize and equip health care teams	
		Use information systems	
		Support self-management and prevention	
**Macro**	Health care organization	Strengthen partnerships	Project director; articles of association; US project director
		Support legislative frameworks	
		Integrate policies	
		Provide leadership and advocacy	
		Promote consistent funding	
		Develop and allocate human resources	

Information was collected using a simple framework based on Table 1 and adapted to the individual or group. Interviews with the project director and head of social work were conducted in English, while those with patients and CPAs at outreach sites were conducted through an interpreter able to translate from English to Wolatinya (the local language) and vice versa. Notes on the interviews were made in English. Additional information was collected from records and legal documents (including clinic registers, network group evaluations, the MFTPA training guide, annual reports, and the articles of association). Information was collected by GD during visits in September 2007 (from the project director and head of social work), March 2008 (from patients and CPAs), and May 2008 (from the project director, representatives of network groups, and the director of the US funding project). EB clarified additional points in July 2008. Formal qualitative analysis was inappropriate for this type of evaluation research, and we report the findings summarized by level and component of the ICCC framework.

## Findings

The findings are presented below organized by level (micro, meso, and macro) and then component (bold subheadings) of the ICCC framework. This structure corresponds to the “building blocks” of the ICCC, indicated by headings and sub-headings in the original report [6]. The characteristics used to describe each component are summarized before presentation of findings relevant to that component from the evaluation. The source of information is indicated in square brackets.

### Micro-Level

At the heart of the ICCC framework are partnership-forming interactions between patients and families, practice teams, and community partners. These interactions are catalyzed by players who are informed, motivated, and prepared; preparedness representing access to the medications, tools, and skills necessary for self-management [6].

The work of the MFTPA is based on the transformation of treated patients into CPAs. CPAs remain in their own community [patients, CPAs], but are fundamental to the practice team, and so become the embodiment of partnerships between patients, the health care team, and the community. CPAs are selected on the basis of adherence with treatment and having been educated to grade 10, 11, or 12 (school leaver's certificate) [project director]. Basic education and treatment for podoconiosis is relatively straightforward (Box 1), thus potential CPAs are given full-time clinical training over a period of one week, and then become responsible for approximately 2,000 patients per annum in their own community [patient registers].

**Box 1. Basic Education and Treatment for Podoconiosis**

Education stresses:

- The agent of podoconiosis (irritant red clay soil)
- The lack of any infectious agent
- Use of footwear and socks to protect against irritant soil

Treatment includes:

- Careful washing with soap and water
- Use of dilute bleach as an antiseptic
- Drying between toes and folds
- Use of moisturizing or antiseptic cream (e.g., Whitfield's ointment)
- Elastic bandaging in selected patients
- Elevation of the leg
- Controlled exercise
- Use of closed footwear and socks

#### Becoming informed

Patients, their families, and the practice team become informed in a two-way process. Patients and their families benefit from CPAs who share their first language (seven languages are spoken in the zone in which the MFTPA works) and can convey education and treatment in easily understandable terms [patients]. The practice team benefits from having patient representatives in its midst, so new treatment or administrative strategies can immediately be checked for practicability in the community setting [project director]. Informing community partners happens mostly through small-scale meetings in schools, churches, mosques, and *kebele* (local administrative area) clubs [head of social work].

#### Becoming motivated

Motivation is boosted as patients see their co-patients become incorporated into the practice team [patients]. Practice team motivation is ensured as the CPAs hold clinics within their own communities and are answerable at a local level for the education and treatment offered [CPAs]. Motivation in the grassroots wider community was harder to assess, but the impression of the project director was that fewer late-stage patients were presenting to the longer established clinics, suggesting that motivation to present for treatment had changed over time.

#### Becoming prepared

Although most of the materials needed for treatment (water, soap, bleach, ointment, socks, and shoes) are simple, many remain out of reach for patients with podoconiosis, who are mostly subsistence farmers with incomes substantially less than US$1 per day [patients]. Preparedness is difficult for patients and families to achieve on their own, thus most rely on the MFTPA for soap, ointment, bleach, socks, and shoes [project director]. The MFTPA tries to assure preparedness by sourcing soap locally, using diluted bleach rather than potassium permanganate (which is not widely available in Ethiopia), and training patients to make shoes (at present, seven patients make approximately 250 pairs of shoes per month in the MFTPA headquarters, and another twenty shoemakers have set up businesses in their own communities) [project director].

### Meso-Level

At this level, the health care organization is important, but the community has a prominent role, and therefore the quality and depth of the organization's interactions with the community are vital.

#### (i) The Community

*Raise awareness and reduce stigma*. Sixty-eight network groups have been established around Wolaita zone [network group register]. They are expected to raise awareness of podoconiosis through (for example) local administrative meetings, school assemblies, church services, and coffee ceremonies (the key social meeting place for women in the zone) [project director, head of social work].

*Encourage better outcomes through leadership and support*. Network groups are evaluated against a framework of activities achieved (for example, number of meetings held) [network group evaluations], but formal assessment of the impact of these activities on attitudes or other longer-term outcomes has not yet been conducted.

*Mobilize and coordinate resources*. Network groups are encouraged to raise funds to assist patients and support services in their area [network group representatives, project director]. However, a rigorous assessment of community resource mobilization has not been made.

*Provide complementary services*. At present, government services for podoconiosis patients are almost non-existent [patients, CPAs, network group representatives]. The MFTPA has attempted to address this gap by training government health workers (nurses and health officers, chiefly), but this has been undermined by rapid turnover of these health professionals within the zone. The long-term strategy of the MFTPA is to assist in development of government services so that podoconiosis patients can be treated independently of MFTPA assistance [project director].

#### (ii) The Health Care Organization

*Promote continuity and coordination*. Podoconiosis patients are highly stigmatized and often face extreme financial hardships, being unable to continue their work as subsistence farmers [head of social work]. The MFTPA understands the importance of making strong links with community organizations including *idirs* (community-based organizations acting like insurance funds in many parts of Ethiopia), churches, mosques, and non-governmental organizations, in order that patients can access a wide range of social support [project director].

As indicated above, coordination with government heath services is weak, chiefly because of the lack of expertise and training of government health professionals in relation to podoconiosis [project director]. Many MFTPA outreach clinics are held on the premises of government clinic sites, but communication between MFTPA workers and government clinic workers is often poor because the CPAs are perceived by health professionals as under-qualified [CPAs].

Onward referral of the small percentage of podoconiosis patients who require surgery (nodulectomy) is well-managed. Clear referral criteria have been agreed upon between the field team and the lead surgeon [project director], and feedback on the long-term results of surgery is passed by the field team back to the surgical team.

*Encourage quality through leadership and incentives*. The MFTPA is supported by a local board of directors, and is headed by a motivated project director, who has previous experience in promoting community participation in the same area [annual report]. Although the director has actively cultivated support at regional, *woreda*, and zonal levels, the organization has little recognition from the Federal Ministry of Health.

*Organize and equip health care teams*. The MFTPA mobilizes their most important resource, podoconiosis patients, by training them as CPAs [project director]. Coordination of services within the MFTPA is good, with quarterly meetings that draw together the clinical, administrative, social work, and community advocacy teams to share good practices and identify important strategy areas [network group representatives, CPAs].

*Use information systems*. The information system used by the MFTPA is paper-based, reflecting the lack of technological expertise among CPAs, of power supplies at most outreach clinic sites, and of personnel at the project base equipped to utilize electronic databases [project director, training guide]. The importance of accurately kept patient registers is understood by CPAs. Each outreach clinic site keeps a register to record sociodemographic and clinical information at presentation, to monitor attendance, to evaluate the effect of treatment, and to document distribution of materials and medications [clinic registers].

*Support self-management and prevention*. Having established that podoconiosis has a strong heritable component [10], the MFTPA has launched a program to provide footwear to 20,000 unaffected children of affected parents [annual report]. CPAs offer primary prevention through education in schools, churches, and women's groups [CPAs, network group representatives]. The MFTPA also encourages secondary prevention through vocational training of treated patients in occupations minimizing contact with irritant soil (hairdressing, carpentry, electronics, bicycle repair, and shoemaking) [patients]. Shoes made by treated patients are supplied at heavily subsidized rates to patients, again contributing to secondary prevention [patients].

### Macro-Level

At present, the MFTPA operates in a policy environment that is far from positive. Disease treatment and control do not feature in undergraduate health professional curricula, and there is no mention of the condition in Ethiopia's Health Sector Strategic Plan. Although the Federal Ministry of Health has given the MFTPA authorization to become the national center for podoconiosis control [annual report], this endorsement is backed with little material support.

#### Strengthen partnerships

The MFTPA has few helpful partnerships, and all are at a relatively local level, with, for example, the Zonal Development and Health Bureaus [project director].

#### Support legislative frameworks

The MFTPA has not yet been involved in attempts to influence legislation in Ethiopia [project director].

#### Integrate policies

At present there is neither a national policy for podoconiosis control nor one for other non-communicable diseases in Ethiopia.

#### Provide leadership and advocacy

The ICCC states that “Decision-makers can influence senior political leaders to advance care for chronic conditions. Political leaders need to be identified and then encouraged to create a positive policy environment for patients, their communities and health care organizations managing chronic problems” [6]. In 2005, Ethiopia's most famous athlete, Haile Gebreselassie, endorsed a mass-participation road race to promote awareness of podoconiosis and reduce stigma associated with the condition. The race was held in the town in which the MFTPA is located and helped draw regional support for the organization [project director].

#### Promote consistent funding

At present, the primary source of funding is the Mossy Foot Project (http://www.mossyfoot.com/). This US-based fundraising trust has its own Executive Board, selected to guide decision-making and raise funds primarily on the west coast of America [director, US funding project].

#### Develop and allocate human resources

The MFTPA has not been involved in development of human resources beyond the CPAs immediately involved in the Association. Allocation is likewise beyond the scope of the MFTPA at present [project director].

## Discussion

We selected the ICCC framework as a useful tool by which to perform an initial evaluation of a chronic disease program in a low-income setting. However, there were certain limitations associated with using this framework. Firstly, the ICCC is narrative, and does not set out indicators suitable for making quantitative assessments. We were therefore not able to “score” the MFTPA, making it difficult to compare the MFTPA with other organizations or with itself over time. However, we were able to identify broad areas in which the organization was deficient, which we anticipate will guide the MFTPA as it expands activities.

Secondly, the absence of a quantitative checklist makes bias more likely. Three types of bias are likely to have influenced this evaluation. The first is a form of selection bias that may have occurred when identifying individuals or groups as information sources. Most of these information sources were identified through the MFTPA, thus we may have interviewed people more likely to give favorable responses. Second, a type of response bias (social desirability bias) may have occurred, particularly when gathering information from patients and CPAs. These groups are very vulnerable, and individuals may have thought that offering criticism might affect their future treatment or employment. We emphasized that the information they gave would not be identifiable at an individual level, but this bias may nevertheless have occurred. Third, it is possible that the investigators introduced bias in recording or interpretation of responses. Wherever possible, we attempted to triangulate information by referring to registers or documents.

The MFTPA was found to be weak with regard to a number of ICCC “components.” These will be discussed, and recommendations made where appropriate.

At micro-level, there is evidence that patients, their families, and the practice team are informed and motivated. Their preparedness is compromised by the extreme poverty in which much of this community lives, making access to even basic materials for treatment and prevention a challenge. Expansion of the MFTPA must take into account the difficulties affected individuals will have in accessing water, soap, ointment, and footwear. Work to develop inter-sectoral partnerships with government and non-government groups active in poverty alleviation and water and sanitation programs will be important in improving preparedness of patients and the practice team families.

Stigma related to podoconiosis, which is one of the factors influencing motivation within the wider community, was investigated in an earlier study, and found to be widespread [11]. The project director's comment that patients were presenting at earlier stages in the longer-established clinics suggests that patients may find it easier to acknowledge presence of disease and access treatment once their community is aware of treatment and prevention options. Formal evaluation of the impact of network groups on levels of stigma in the community is recommended.

Although there are enormous benefits in using patients as health agents, resource constraints mean that the extent of training given to CPAs is limited and external oversight is minimal. Coordination between CPAs and existing government health services is still weak, so podoconiosis patients with dual pathology are poorly served, since CPAs have insufficient training to identify and treat other diseases. Better use of CPAs could be made given a basic external supervision structure and a stronger referral system. This might be achieved through, for example, a monthly “difficult cases” training clinic at which a dermatology nurse or dermatologist could review patients in the presence of their CPA.

Although the manual clinic registers are adequate for the relatively modest demands of tracking patient attendance and response to treatment, they are difficult to combine to make an overall assessment of MFTPA activities and plan future care. There are difficulties related to using more sophisticated technology in the remote areas in which patients live. The Ethiopian government has ambitious plans to extend electricity supplies and Internet connections down to the “*woreda*” (second lowest unit of administration) level. This may in future enable creation of a simple central electronic database to which data may be added from each outreach clinic site.

In order to expand its activities beyond Wolaita zone, the MFTPA will need to develop partnerships with groups active at the community level in other zones and regions of Ethiopia. For example, the trachoma and onchocerciasis control initiatives of the Carter Center (http://www.cartercenter.org/) in Ethiopia have well-developed community networks in several podoconiosis-endemic areas, and rather than duplicating these, the MFTPA might partner to develop community conversations about podoconiosis through them. World Vision (a non-government organization emphasizing multisectoral development in specific area development programs) also has community networks in several of the areas into which the MFTPA is looking to expand.

At present, the MFTPA lacks leadership able to influence national policy on podoconiosis. Several commentators have identified the importance of powerful leadership figures in generating political priority for a condition or initiative [8],[12]. “Special ambassadors” for particular causes can help create awareness about a condition, and create the environment within which senior policy makers then develop favorable policy. Identification of such leadership figures is an urgent priority for the MFTPA in order that nationwide interventions affecting podoconiosis care in education, training, and health care provision are developed.

The MFTPA has not to date been able to influence the content of pre-service education and training. Basic information on the treatment and control of podoconiosis must be included in the pre-service training of many professionals, including medical students, nurses, health officers, environmental health officers, public health officers, health extension workers, water engineers, and agricultural scientists. In-service training must be developed for health, water and sanitation, and agricultural professionals in all five Ethiopian regions with a significant podoconiosis burden.

The US Mossy Foot Project has been vital to the establishment and development of the MFTPA, and still raises over 90% of the funding necessary to run activities. However, this group cannot ensure consistent funding at a level to support expansion of the MFTPA throughout Ethiopia. Long-term investment in health infrastructures will be necessary, and political commitment to support the expansion of the MFTPA through direct funding and in kind will be essential.

## Conclusions

We used the ICCC framework to make a constructive review of a non-government organization in a low-resource setting. Working systematically through components at micro-, meso-, and macro-levels enabled us to identify operational areas that are functioning well, others that are under-performing, and others not yet attempted.

Despite the extremely poor community and patient group in which the MFTPA operates, it is strong at micro-level. This is chiefly because of the MFTPA practice of transforming patients into CPAs who almost by definition encourage partnership-forming interactions between patients and families, practice teams, and community partners.

CPAs become channels of information and models of motivation for the community. As treated patients, they are motivated, and as part of the health care team they are prepared with the basic materials required for prevention and treatment. In future, optimal use of CPAs will be achieved through more thorough supervision and an improved system of referral for coexisting health problems.

The MFTPA effectively uses community groups to advocate for podoconiosis control and to reduce stigma against podoconiosis. Development of these “network groups” is relatively recent, and their true impact on resource mobilization and stigma reduction has not yet been formally assessed. Coordination with zonal government health services is currently weak because the MFTPA is the only organization providing podoconiosis treatment and control. Earlier attempts to train government health workers in podoconiosis management must be strengthened.

The MFTPA is a grassroots organization that was established only ten years ago. Unsurprisingly, it is still relatively ineffective at macro-level within Ethiopia or on the international stage. Evaluation of current strategies against macro-level ICCC components suggests that the most important goals for the near future will be to develop and strengthen partnerships with other organizations that have effective community networks; to identify powerful advocates who can influence senior policy makers; and to encourage sustainable sources of funding from within Ethiopia. Only with these components in place can the MFTPA hope to achieve expansion of podoconiosis control through Ethiopia.
